# Automated in-situ monitoring of accelerated crystallization processes of nifedipine using terahertz time-domain spectroscopy

**DOI:** 10.1038/s41598-024-81316-y

**Published:** 2024-12-02

**Authors:** Lara Heidrich, Julian Wiener, Enrique Castro-Camus, Martin Koch, Jan Ornik

**Affiliations:** 1 Department of Physics and Material Sciences Center, Philipps-Universität Marburg, Renthof 5, 35032 Marburg, Germany; 2 Centro de Investigaciones en Optica A.C, Loma del Bosque 115, Lomas del Campestre, Leon, Guanajuato, 37150 Mexico; 3 Leibniz Institute of Photonic Technology, Member of the Research Alliance, Leibniz Health Technologies, 07745 Jena, Germany; 4 Cluster of Excellence Balance of the Microverse, Friedrich Schiller University Jena, 07743 Jena, Germany

**Keywords:** Terahertz spectroscopy, Automatization, Quality control, Crystallinity, Pharmaceuticals, Applied physics, Optics and photonics, Pharmaceutics

## Abstract

We developed and tested an automated measurement platform which can fit multiple samples for their investigation in transmission mode using terahertz time-domain spectroscopy. The temperature inside the platform can be varied to simulate different storage conditions of the samples, in our case, pharmaceuticals. As a proof-of-concept, the setup was successfully tested to monitor the crystallization process of amorphous nifedipine, as a model drug, at 24 °C, 30 °C and 35 °C for over 144 h. To the best of our knowledge, this is the first study to follow the crystallization of nifedipine with quasi-continuous measurements over a time frame of several days. The influence of the storage temperature on the crystallization rate was monitored including the appearance of polymorphic intermediate states of nifedipine throughout the process. The platform developed in combination with terahertz time-domain spectroscopy is a helpful tool for deepening the understanding of the crystallization behavior of amorphous and polymorphic materials and can be, for example, of great importance for the development of novel amorphous pharmaceutical formulations.

**Supplementary Information:**

The online version contains supplementary material available at 10.1038/s41598-024-81316-y.

## Introduction

Crystallization processes appear both in nature, for instance, in the formation of ice or bones, as well as in industry or research, for example in the manufacturing or purification of various materials^1^. Surprisingly, despite its significance, the underlying mechanisms of nucleation and crystal growth on the molecular-scale are still not fully understood and are part of ongoing research^2–5^. Also in the pharmaceutical community, the control and modification of the solid state of an active pharmaceutical ingredient (API) has gained significant attention^6^. Different crystalline forms of an API can show different water solubility which, in turn, can affect the bioavailability after oral administration^7^. Usually, amorphous APIs have a higher water solubility than their crystalline counterparts^7^. This is of enormous importance as many newly developed APIs exhibit poor water solubility which could be enhanced by administrating them in an amorphous form^8^. In such cases, however, careful quality control is mandatory to ensure proper bioavailability due to the intrinsic instability of most amorphous APIs and the resulting risk of recrystallization^9^.

In order to gain a deeper understanding of the stabilization mechanism, e.g. in amorphous solid dispersions or co-amorphous systems, systematic isothermal long-term studies are needed^10^. When dealing with the stability of amorphous API systems, it is generally assumed that the main factor determining nucleation and crystal growth is the primary diffusive molecular mobility of the glassy state as a function of temperature^11^. Thus, studying the temperature-dependent crystallization from a glassy state is of fundamental importance^11^. Yet, such systematic studies can result in high workload and material-consumption. For instance, if a destructive analysis technique is used, several batches are required.

One technique that has proven to be suitable for non-destructive crystallinity assessment is terahertz (THz) time-domain spectroscopy (TDS)^12^. The THz band covers the frequency range from 0.1 to 10 THz and falls between the microwave and infrared regime^13^. In recent years, various possible applications of THz systems have been presented, such as telecommunications^14^, medical diagnostic^15^or the analysis of cultural heritage^16^. A detailed and extensive overview on THz technology and further fields of application can be found for instance in works by Neu and Schmuttenmaer^17^ or Leitenstorfer et al.^18^. Using THz-TDS, non-covalent interactions – such as phonon modes in small molecular crystals – can be probed directly^19^. This leads to distinct absorption peaks in the THz range for crystalline substances, whereas amorphous materials only show a featureless monotonously increasing absorption spectrum as a function of the frequency^20^. Consequently, THz-TDS is highly sensitive to changes in the crystal structure and can be used for polymorph identification^21^. The applicability of THz-TDS to study the crystallinity was already demonstrated for several APIs^22–28^as well as for the investigation of the crystal nucleation and growth from solution^5,29^.

Although versatile applicability has been demonstrated successfully in recent years, THz-TDS is still a method mostly used by specialists. Operating the systems, handling and positioning of the samples as well as interpreting the measurement data often requires trained personnel. For wider acceptance, operation of THz-TDS devices must become easier, so they can be operated by non-technically trained laymen^13^. Hence, further development of hardware is required that is not only easy to operate and precise, but also more cost-effective and more robust than the systems nowadays. This includes not only THz-TDS systems itself but also user-friendly sampling platforms, such as handheld instruments or sample chambers.

In this work we designed, constructed and tested a new automated, temperature-controllable measurement platform for THz-TDS in transmission mode. The goal was to build a platform that allows for the systematic investigation of batches of samples, in this case bi-planar tablets representing typical pharmaceutical dosage forms. The storage temperature is controllable to simulate accelerated aging conditions. Furthermore, to enable the variable application and implementation into tight spectroscopy setups, the platform can be easily adjusted to other sample geometries and its design aims to be robust and space-saving in combination with variable mounting options. In addition, the measurements are automated to keep the workload as low as possible.

As a pharmaceutical model system, nifedipine (NIF) was chosen. NIF is a calcium-channel blocker and exhibits poor solubility in water^30^. Its polymorphism has been studied for more than three decades^31^and it has been used as an amorphous model drug in several studies^30,32–36^. Moreover, NIF has already been studied using THz-TDS^37–39^. Additionally, the recrystallization process of neat amorphous NIF has been investigated in previous works for timeframes ranging from a few hours up to three days^34,40,41^. Herein, we examined the recrystallization of amorphous NIF samples as a proof-of-concept for 144 h at 24 °C, 30 °C and 35 °C by using the measurement platform developed.

## Results

### Automated measurement platform

**Fig. 1:**
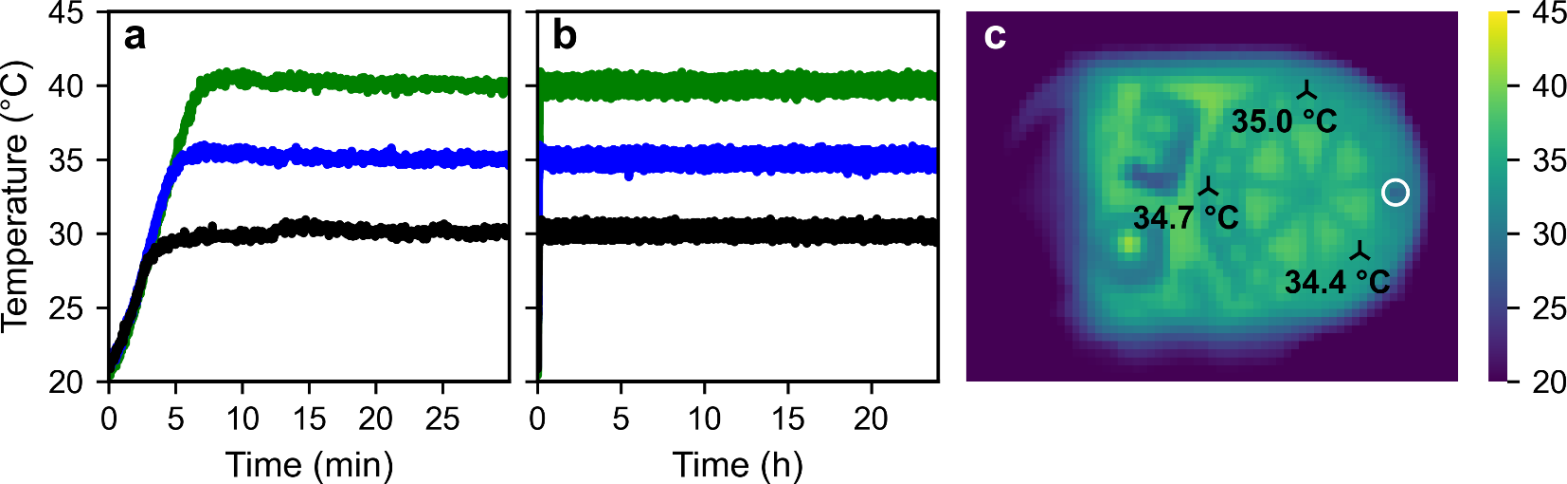
(**a**) Heat-up phase and (**b**) temperature-stabilization inside the T-Box over 24 h for three target temperatures (black 30 °C, blue: 35 °C and green: 40 °C; each averaged over the two installed temperature sensors). (**c**) Thermal image of the sample chamber immediately after opening the T-Box with a target temperature of 35 °C and annotated temperatures of specific parts (black triangles). The white circle indicates the measurement aperture where the samples during measurements are located.

First, the performance of the temperature-controllable measurement platform, the so-called T-Box^42^, was tested. In Fig. 1 the temperature logged during the heat-up-phase (Fig. 1a) as well as over 24 h (Fig. 1b) is shown for three target temperatures. Inside the T-Box, the desired target temperature can be reached in less than 10 min (Fig. 1a). Furthermore, a constant temperature with an absolute deviation of less than 1.5 °C can be maintained (Fig. 1b). In Fig. 1c, the heat distribution of the sample chamber immediately after opening the T-Box with a target temperature of 35 °C is shown, confirming a homogenous distribution across all samples.

### Polymorphism of nifedipine

**Fig. 2:**
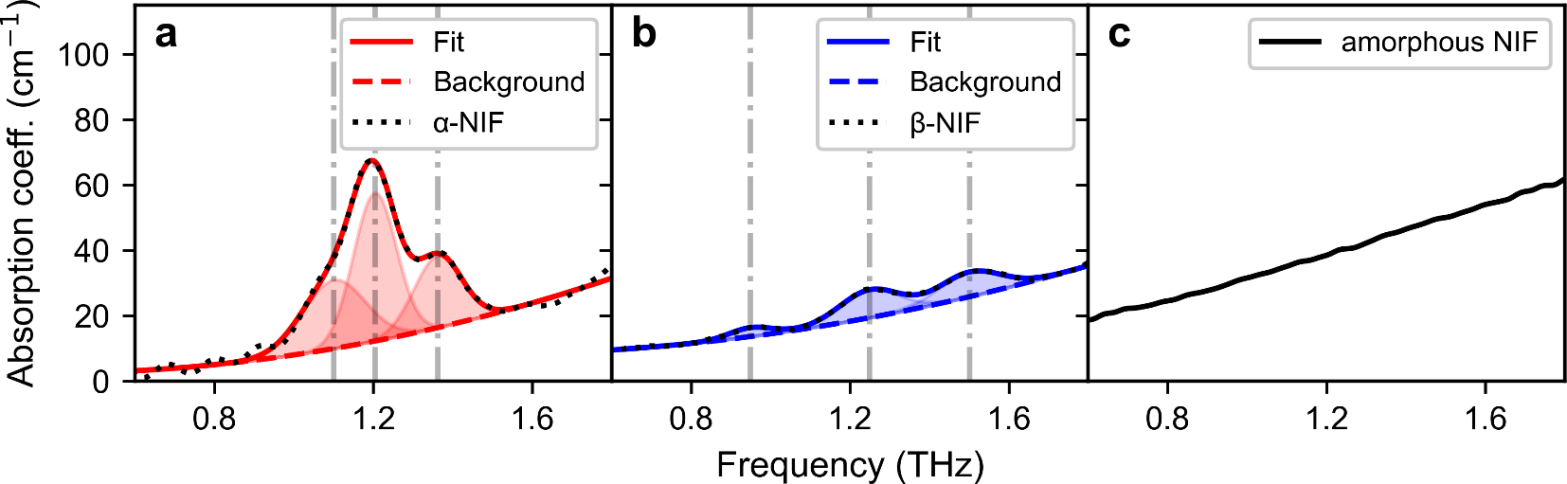
THz absorption coefficient (coeff.) spectra of (**a**) α-NIF, (**b**) β-NIF and (**c**) unaltered amorphous NIF. In (**a**) and (**b**), the black dotted line corresponds to the original spectrum, the solid line to the fitted spectrum and the dashed line to the monotonously increasing background absorption. The vertical grey dashed-dotted lines mark the frequency positions of the identified absorption peaks (shaded area).

In this work, we focused on three forms of NIF: first, the stable α-crystalline form, second, the metastable β-crystalline form and third, an unstable amorphous form. The THz absorption spectra of the three forms are shown in Fig. 2.

Regarding the α-form, three peaks centered at 1.09 THz, 1.20 THz and 1.36 THz are found, whereas for β-NIF three weaker peaks located at 0.95 THz, 1.25 THz and 1.50 THz can be identified. On the contrary, amorphous NIF only shows a monotonously rising absorption, which is typical for disordered materials^43^. Thus, the analysis by THz-TDS allows fast differentiation between the three forms. The absorption peaks of the crystalline forms can each be described quantitatively as Gaussian functions^12^ (red/blue shaded area in Fig. 2a-b). In the following, this will be helpful for advanced crystallinity analysis of aged amorphous NIF samples. For this, the absorption spectra of α- and β-NIF are each fitted by the combination of a sum of the three absorption peaks – represented by Gaussian functions – and a second order polynomial. The second order polynomial is used to describe the increasing featureless background absorption (Fig. 2a-b, dashed lines) which is mainly caused by scattering. Further details on the extended analysis including the extracted peak parameters as well as the application to samples with known NIF-content can be found in the Supporting Information.

### Crystallization studies at controlled temperature

**Fig. 3:**
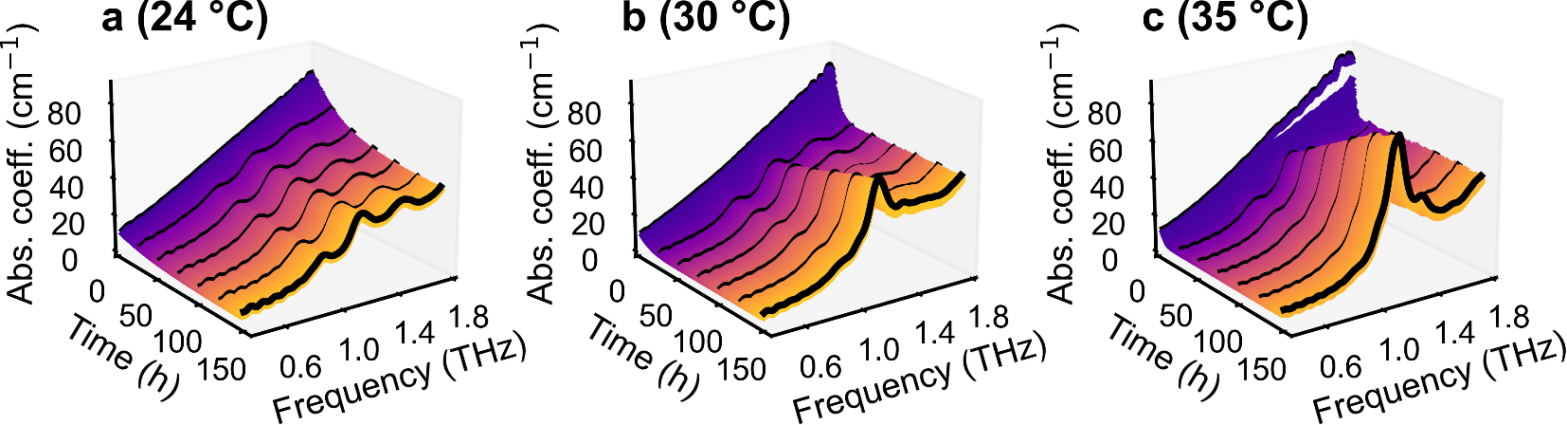
Time-dependent absorption (Abs.) coefficient (coeff.) spectrum of an initially amorphous NIF sample stored at (**a**) 24 °C, (**b**) 30 °C and (**c**) 35 °C. For clarity, every 4th measurement is shown, resulting in steps of approximately 14 min. Curves corresponding to approximately 0, 1, 2, 3, 4, 5 and 6 days are depicted as a black bold curve and plotted with an offset of + 2 cm^−1^ for easier comparison. In total, five samples per storage temperature were examined, showing similar results.

After characterizing the polymorphism of NIF, initially amorphous NIF samples were measured for six days using the T-Box at three different temperatures: 24 °C, 30 °C and 35 °C. The temporal evolution of the absorption spectra of these samples is shown in Fig. 3.

For all samples, first only a monotonously featureless absorption can be seen, indicating a successfully prepared amorphous form. A few hours after starting the measurement the baseline absorption drops and three absorption peaks corresponding to βNIF arise, indicating the crystallization from an amorphous form to the metastable β-form. The decrease of the absolute absorption coefficient with increasing crystallinity can be explained by the collapse of the so-called vibrational density of states and the emerging of coherent vibrations at discrete frequencies^21^. After this intermediate step, the formation of three stronger peaks corresponding to α-NIF can be observed for the samples stored at 30 °C and 35 °C, indicating the conversion to the stable α-form. For the samples stored at 24 °C, this last step of the process is not visible in the time frame investigated. Furthermore, by comparing the temporal evolution of the absorption coefficient at 30 °C and 35 °C, it can be observed that the crystallization of amorphous NIF to the metastable β-form as well as the recrystallization to the stable α-form occurs faster at higher temperatures.

**Fig. 4:**
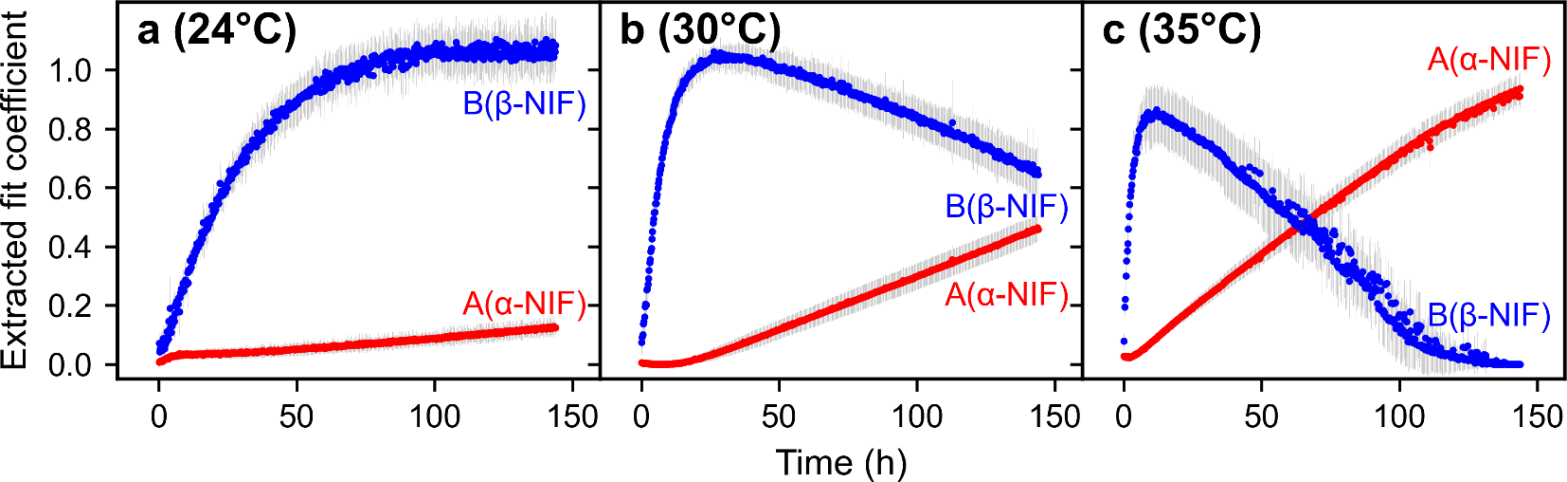
Time-dependent extracted fit coefficients for α-NIF (red markers) and β-NIF (blue markers) of initially amorphous NIF samples stored at (**a**) 24 °C, (**b**) 30 °C and (**c**) 35 °C. The grey error bars represent the standard deviation of the assessed fit coefficients of the five samples investigated per storage temperature. Every 4th measurement was evaluated, which corresponds to steps of approximately 14 min.

In order to gain further understanding of the crystallization, advanced crystallinity analysis was performed, according to Ornik et al.^12^. For that, the extracted peak parameters (see Supporting Information) of the crystalline forms were used and the

1 $$\:f\left(v\right)=A{\sum\:}_{i=1}^{3}{G}_{{\alpha\:}_{i}}{e}^{{-\left(\frac{v\:-\:{v}_{0}^{{\alpha\:}_{i}}}{\varDelta\:{v}_{{\alpha\:}_{i}}}\right)}^{2}}+B{\sum\:}_{i=1}^{3}{G}_{{\beta\:}_{i}}{e}^{{-\left(\frac{v\:-\:{v}_{0}^{{\beta\:}_{i}}}{\varDelta\:{v}_{{\beta\:}_{i}}}\right)}^{2}}+O\left(2\right)$$

function was applied to the time-dependent absorption spectra. The first summation term includes Gaussian peaks related to α-NIF and the second summation term corresponding to β-NIF. Hereby, $\:{G}_{{{\alpha\:}_{i}/\beta\:}_{i}}$, $\:{v}_{0}^{{{\alpha\:}_{i}/\beta\:}_{i}}$ and $\:{\varDelta\:\nu\:}_{{{\alpha\:}_{i}/\beta\:}_{i}}$ correspond to the amplitude, central frequency and width of the $\:i$-th peak of α- or β-NIF, respectively. The $\:O\left(2\right)$-term represents the second order polynomial used to account for the monotonically rising background absorption at increasing frequencies. The fit coefficients $\:A$ and $\:B$ are obtained by fitting the absorption spectra and are related to the α- or β-content in the samples. In Fig. 4 the time-dependent extracted fit coefficients for α- and β-NIF are summarized.

The results of the advanced crystallinity analysis are in line with the previously discussed qualitative findings. Figure 4 reveals that for all three storing temperatures the samples were initially mainly amorphous. In case of the lowest temperature (Fig. 4a), an immediate increase of the fit coefficient $\:B$ corresponding to the β-form can be observed, indicating the onset of crystallization. After approximately 100 h, the fit coefficient $\:B$ reaches a plateau whereas a slow increase in the fit coefficient $\:A$ corresponding to the α-NIF content can be observed. For samples stored at 30 °C (Fig. 4b) the formation of β-NIF occurs faster with the fit coefficient $\:B$ reaching the peak value already after approximately 27 h. In the following, the value of the fit coefficient $\:B$ starts decreasing, whereas at the same time the value of the fit coefficient $\:A$ increases. This indicates the conversion of the metastable β-form to the stable α-form. Again, also the increase of the fit coefficient $\:A$ appears faster for the samples stored at 30 °C compared to those stored at 24 °C. An even faster recrystallization to β-NIF and subsequent transformation to α-NIF can be found regarding the samples stored at 35 °C (Fig. 4c). In this case, the fit coefficient $\:B$ reaches its peak value already after approximately 12 h. Then, both the decrease of the fit coefficient $\:B$ as well as the increase of the fit coefficient $\:A$ occur faster, when compared to the crystallization processes at 24 or 30 °C. This denotes an even faster conversion of β-NIF to the stable α-form.

In summary, for all storage temperatures, the samples are initially mainly amorphous and then crystallize with an intermediate step via the metastable β-form, followed by subsequent formation of the stable α-form. Both the crystallization to β-NIF and the conversion to α-NIF occur faster at higher temperatures.

## Discussion

The investigation of crystallization and aging processes is an important factor in the manufacture and use of amorphous pharmaceutical formulations^10,34^. However, systematic long-term studies can be time-consuming, material and labor-intensive, even more if several batches are needed. The developed measurement platform enables the automated temperature-dependent investigation of several samples. Thus, samples manufactured by different preparation methods or with different additives can be investigated under identical storage conditions, leading to further insights into the physical stability of amorphous APIs. The platform can be easily integrated into a typical THz-TDS setup and – due to the fabrication of the T-Box by 3D printing – it can be quickly adapted to new sample geometries. So far only a small storage temperature range can be set using the T-Box (range: ambient temperature to 45 °C). However, this region corresponds to the typical temperatures to which pharmaceutical products may be exposed, for instance during transport. Yet, the temperature range could be extended with minor adjustments, for instance, by using more heat-resisting materials or by including active cooling. In addition, to the best of our knowledge, so far only measurement platforms have been used that allow either the temperature-independent analysis of several samples or the temperature-dependent analysis of a single sample. Hence, the T-Box represents a unique tool for analyzing multiple samples under controllable temperature using THz TDS in transmission mode.

As a proof-of-concept, the effect of the storage temperature on the crystallization of NIF has been successfully demonstrated. Hereby, amorphous NIF first crystallizes to metastable β-NIF and then converts to the stable α-form. This process occurs faster at higher storage temperatures and could also be analyzed in an advanced manner. In general, these findings agree with previous studies and have been reported earlier^34^. However, usually differential scanning calorimetry^44^, x-ray powder diffraction^45^or optical spectroscopy^46^have been used to determine the crystallinity of pharmaceuticals. In contrast, the use of THz-TDS can offer a unique perspective to study crystallization processes^29^due to its non-destructive nature, the fast data-acquisition and high ability to directly probe intermolecular vibrations. Moreover, previous studies followed the crystallization of NIF either with short measurements intervals (i.e. of a few minutes) for a few hours up to three days^34^^,40 ^or with long measurement intervals (i.e. several days or weeks) for weeks to months^37^^,47^. Hence, to the best of our knowledge, this is the first study investigating the crystallization of amorphous NIF for more than 100 h with comparatively short intervals between the measurements.

With regard to the application of amorphous APIs, our experiments can also be interpreted as a simplified simulation of everyday scenarios in the transport and storage of pharmaceutical products. For instance, tablets containing an amorphous drug could experience undesirable temperature fluctuations between its fabrication and use at home, which, in turn, may affect the quality of the medicine. Another example is forgetting or storing pharmaceutical products in a car on hot summer days, resulting in storage temperatures similar to the experimental conditions shown in this work. In the case of amorphous NIF, exposing it to elevated temperatures leads to a faster crystallization to a less soluble form. Consequently, this can lead to decreased oral bioavailability and significant problems with respect to effective oral drug delivery.

## Conclusions

In summary, a cost-effective and automated measurement platform was developed for long-term, in-situ THz-TDS investigation of multiple samples. The platform can be easily integrated into typical setups and allows for the autonomous monitoring of several samples at a desired temperature. The capability to non-destructively monitor the crystallization of amorphous NIF was demonstrated. Furthermore, it was possible to observe polymorphic transitions of NIF with our setup. Our results indicate the promising potential of the T-Box to study further accelerated aging processes in-situ at increased storage temperatures, for instance, of pharmaceutical formulations. It could be used to gain a better understanding of crystallization processes or stabilization mechanisms in amorphous or polymorphic materials with minimal manual effort and minimal material consumption. Future work should focus on expanding the storage conditions inside the T-Box, for example, by expanding the possible temperature range and by including a humidity control unit. Furthermore, the T-Box should be used for comparative stability studies of different amorphous pharmaceutical formulations.

## Experimental section

### Automated measurement platform

**Fig. 5:**
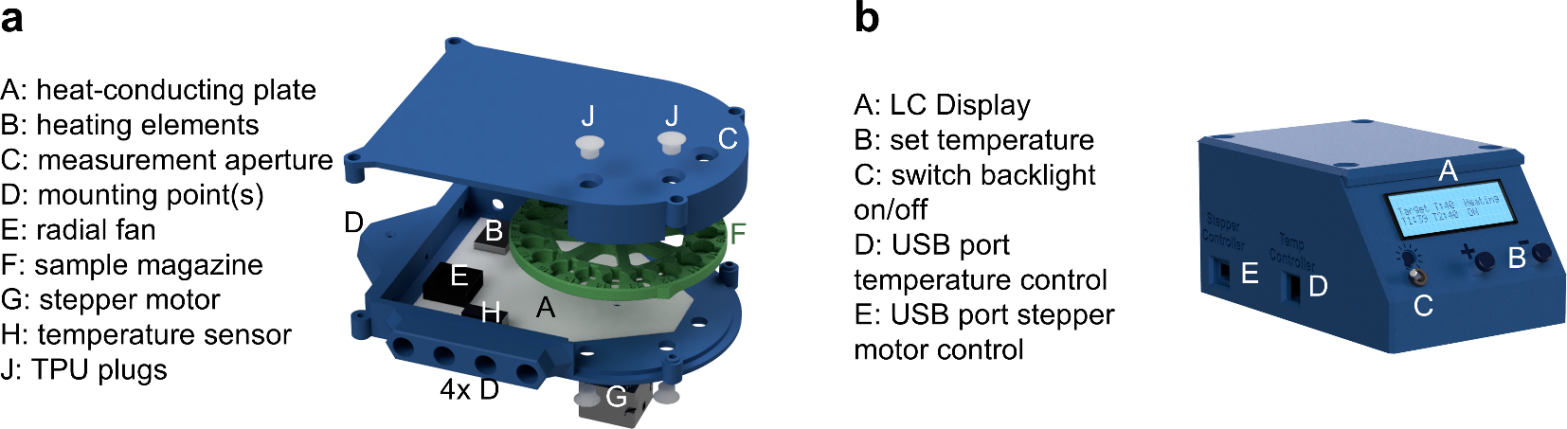
**(a)** Design of the developed measurement platform consisting of a sample chamber and a sample magazine as well as its control unit **(b).**

The temperature-controllable measurement platform, the so-called T-Box^42^, consists of three main parts: a sample chamber, a sample magazine as well as an external control unit. The design of the developed prototype including labeled component parts is shown in Fig. 5.

The sample chamber (Fig. 5a), sample magazine (Fig. 5a, F) and control unit (Fig. 5b) were first designed using a computer aided design program and then manufactured by fused deposition modeling 3D printing. This enables rapid prototype iterations as well as subsequent adaptations, for example to new sample geometries. Polyethylene terephthalate glycol was chosen as the printing filament for the sample chamber and magazine due to its higher temperature resistance. Flexible plugs (Fig. 5a, J) 3D-printed from thermoplastic polyurethane (TPU) are used to fix the sensitive samples in the sample positions. The magazine is reinforced by an aluminum insert at the attachment point to the axis of rotation and can be moved via a stepper motor (Fig. 5a, G) for sample positioning. The T-Box can be operated from ambient temperatures up to 45 °C. This is realized by a proportional-integral-differential temperature controller, two temperature sensors (Fig. 5a, H), a microcontroller and two heating resistors (Fig. 5a, B). An active radial fan (Fig. 5a, E) and aluminum plates (Fig. 5a, A) are used for homogeneous heat distribution. In addition, an insulation layer is attached to the outside of the T-Box for better temperature stability and lower energy consumption. The maximum internal temperature of the T-Box could be further increased by installing additional heating elements, improved thermal insulation and the use of more heat-resistant materials. Three measurement holes (Fig. 5a, C) and five mounting options (Fig. 5a, D) enable variable installation into typical transmission setups.

An external control unit (Fig. 5b) is used to set the target temperature (Fig. 5b, B). The control unit is relocated to an extra housing so that the T-Box could also be installed in tight setups. The cables for the motor, the heater and the measuring probes each have their own cable harnesses to avoid interferences (Fig. 5b, D-E). All units have different interfaces to prevent incorrect connection. The control unit also has a display (Fig. 5b, A) that shows the target temperature and the measured temperature of the two measuring probes, as well as whether the sample chamber is currently being actively heated via the resistors or not. This heating and temperature information is exported every second via a serial interface. The display backlight can be switched off for light-sensitive setups or experiments (Fig. 5b, C). A sample can be selected and measured directly via the measuring software using an interface (mini-USB port) with the measuring computer.

To check whether the self-developed measurement platform works as intended, preliminary tests were carried out. For this, three test target temperatures (30 °C, 35 °C and 40 °C) were set and the heat-up phase as well as the temperature stabilization over 24 h inside the sample chamber was monitored by the two sensors. Furthermore, the heat distribution and possible heat bridges were checked by using a thermal camera (Voltcraft, WBP-120).

### Sample preparation

As a model system, amorphous NIF tablets were studied. In general, it is known that NIF shows polymorphism with α-NIF as the most stable form^31,48^. For the preparation of amorphous NIF, commercially available α-NIF (abcr, 98%) was transferred to an amorphous form by melt-quenching. Therefore, α-NIF powder was placed in an aluminum pan on a hot plate and heated up to 183 °C until it completely melted. Afterwards, the melt was cooled down quickly to room temperature. The resulting glass was ground carefully and pressed to form tablets using a hydraulic press (Enerpac, GF-10B Cl. 1.0). For this a compression force of 15 kN was applied for 90 s. The thickness was determined using a micrometer screw and the samples were stored briefly in a fridge (4 °C) before being inserted into the sample wheel. All tablets had a diameter of 13 mm and a thickness of 1.1–1.6 mm.

In addition, β-NIF, a metastable polymorph, was prepared by storing an amorphous sample at 40 °C for four days in a desiccator over silica. Furthermore, a tablet based on bulk α-NIF was pressed. The polymorphism of NIF was controlled via x-ray powder diffraction (see Supporting Information). The entire sample preparation was carried out under yellow light conditions to prevent the photochemical degradation of NIF^49^.

## Terahertz time-domain spectroscopy

**Fig. 6:**
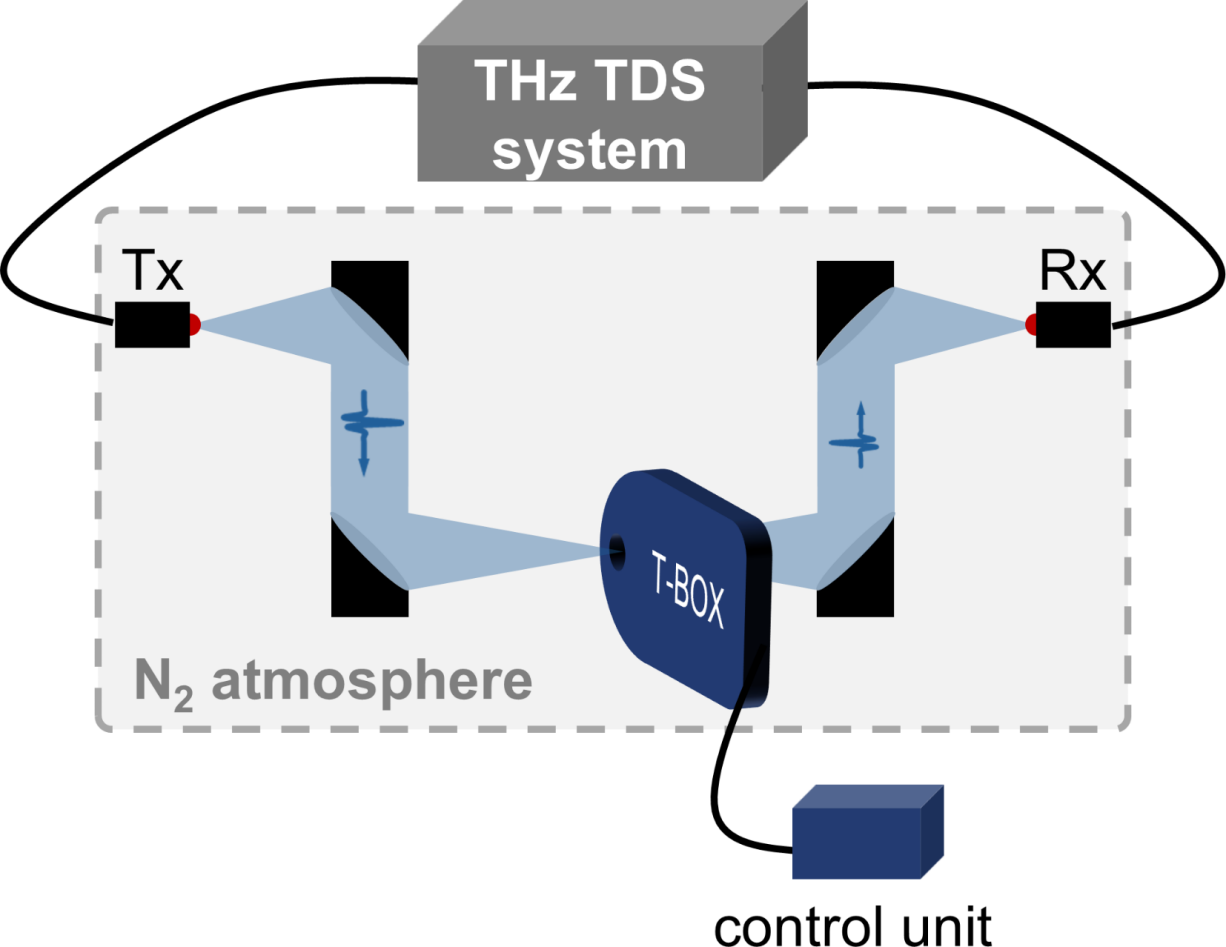
Schematic implementation of the T-Box into the fiber-coupled THz-TDS setup (Tx: transmitter antenna, Rx: receiver antenna).

A fiber-coupled THz-TDS system^50^ with a focused beam in a transmission geometry was used for the crystallinity analysis. To minimize the absorption of THz radiation by the water vapor in the ambient humid air, all THz-TDS measurements were conducted in a plastic box under nitrogen atmosphere. For the aging studies, all samples were fixed in the sample magazine. Then, the sample magazine was put inside the T-Box which was in turn placed in the THz-TDS setup. The sample magazine was aligned in the THz setup so that the samples could be measured successively in the focal plane. For this, the time-resolved waveform of a THz electromagnetic transient passing through a sample was recorded and averaged over a total of 100 measurements. Reference measurements of nitrogen were performed by selecting an empty slot of the sample magazine. Additionally, measurements of nitrogen with and without the T-Box installed were carried out to estimate the influence of the T-Box within the setup on the THz-TDS results. Hereby no significant influence on the measurement was found (see Supporting Information). A scheme of the implementation of the T-Box can be found in Fig. 6.

Initially, samples made from α-NIF, β-NIF and unaltered amorphous NIF were measured. Five measurements, each with 100 averaged waveforms were recorded for every tablet.

The individual acquired time-resolved THz waveforms were first preprocessed by removing the DC offset through subtracting the mean amplitude of the first 5 ps. Furthermore, a Tukey-window and zero-padding was applied followed by a Fourier-transformation to the frequency-domain. Further details about the preprocessing of THz-TDS data can be found in the Supporting Information. In addition, a detailed description of the background of the individual processing steps can be found, for instance, in a review article by Koch et al.^13^. The absorption coefficient $\:\alpha\:$ was then determined via the refractive index $\:n$ according to Withayachumnankul and Naftaly^51^. In short, the absorption spectra were calculated by

2 $$\:\alpha\:\left(\nu\:\right)=\:-\:\frac{2}{d}ln\left(\frac{{\left(n\left(\nu\:\right)+1\right)}^{2}}{4n\left(\nu\:\right)}\left|\stackrel{\sim}{T}\left(\nu\:\right)\right|\right)$$

with

3 $$\:\left|\stackrel{\sim}{T}\left(\nu\:\right)\right|=\:\left|\frac{{\stackrel{\sim}{E}}_{sample}}{{\stackrel{\sim}{E}}_{reference}}\right|$$

and

4 $$\:n\left(\nu\:\right)=\:\:1+\:\frac{c{\Delta\:}\phi\:\left(\nu\:\right)}{2\pi\:\nu\:d},$$

hereby $\:\nu\:$ represents the frequency, $\:d$ the sample thickness, $\:\stackrel{\sim}{T}\left(\nu\:\right)$ the transfer function, $\:{\stackrel{\sim}{E}}_{sample}$ and $\:{\stackrel{\sim}{E}}_{reference}$ the Fourier-transformed electric fields of the sample and reference measurement, $\:c$ the speed of light and $\:{\Delta\:}\phi\:$the difference between the processed phase of the Fourier-transformed sample and reference signals^52^.

### Monitoring of aging studies

In total, three individual aging studies with three different storage temperatures were conducted using the T-Box. After positioning the sample chamber including the magazine and samples in the THz setup, the target temperature inside the chamber was set to 24 °C (no heating), 30 or 35 °C. The samples were monitored sequentially for 144 h and each sample was measured every 3.5 min. In total, five identical samples per storage temperature were considered. Due to the large amount of data which needs to be processed, only every 4th measurement was evaluated, corresponding to intervals of 14 min.

## Electronic supplementary material

Below is the link to the electronic supplementary material.

Supplementary Material 1

## Data Availability

The datasets generated and analysed during the current study are available from the corresponding author on reasonable request.
