# Supplementary material for: Automated in-situ monitoring of accelerated crystallization processes of nifedipine using terahertz time-domain spectroscopy
Source: Sci Rep. 2024 Dec 2;14:29928. doi: 10.1038/s41598-024-81316-y (PMC11611916; doi:10.1038/s41598-024-81316-y)
Supplement: Supplementary file 1 — Supplementary Material 1 [file 41598_2024_81316_MOESM1_ESM.pdf]

Supporting Information

Automated in-situ monitoring of accelerated crystallization processes of nifedipine using terahertz time-domain spectroscopy

Author list: Lara Heidrich[1,*], Julian Wiener[1,*], Enrique Castro-Camus[1,2], Martin Koch[1], Jan Ornik[1,3,4]

[1]Department of Physics and Material Sciences Center, Philipps-Universität Marburg, Renthof 5, 35032 Marburg, Germany
[2]Centro de Investigaciones en Optica A.C., Loma del Bosque 115, Lomas del Campestre, Leon, Guanajuato, 37150, Mexico
[3]Leibniz Institute of Photonic Technology, Member of the Research Alliance, Leibniz Health Technologies, 07745 Jena, Germany
[4]Cluster of Excellence Balance of the Microverse, Friedrich Schiller University Jena, 07743 Jena, Germany

*these authors contributed equally

Table of Contents

1. Preprocessing of the THz-TDS data 2
2. Polymorphism of nifedipine: x-ray powder diffraction 2
3. Influence of the T-Box on the THz-TDS measurements 3
4. Advanced crystallinity analysis 3
References 4

1. Preprocessing of the THz-TDS data

An example of the preprocessing of the THz-TDS data regarding a sample measurement is shown in Figure S1 a. The THz waveform was first processed by removing the DC offset through subtracting the mean amplitude of the first 5 ps. Then, a Tukey-window (α = 0.25) was applied to minimize the effect of Fabry-Perot pulses on the analysis in the frequency domain. Details on the effect of windowing on the THz-TDS signal can be found for instance in a work by Vázquez-Cabo and collegues[1]. Subsequently, the signal was zero-padded to its original signal length, followed by a Fourier-transformation to the frequency-domain. The corresponding amplitude spectra of a corrected reference and sample measurement are shown in Figure S1 b.

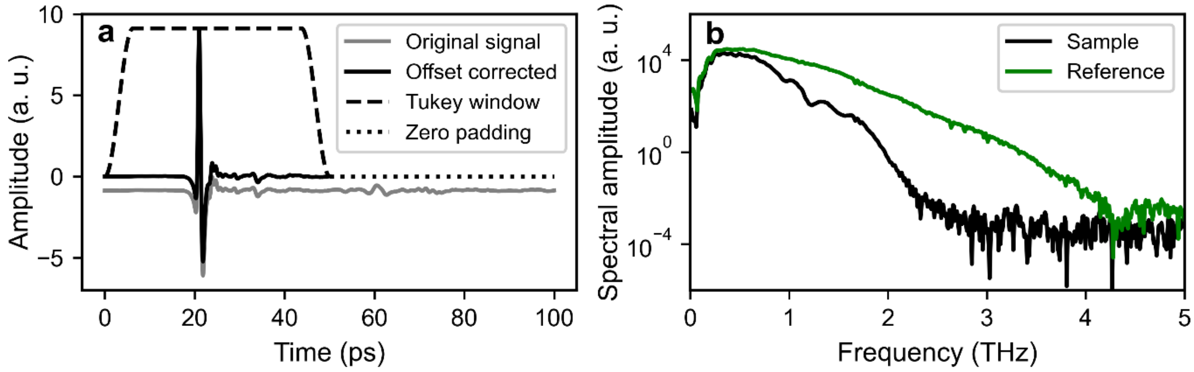

Figure S1. (a) Processing of the THz time-domain signal of a sample measurement. For representation purposes, the values of the Tukey window function (α = 0.25) were multiplied with the maximum value of the offset-corrected waveform. (b) Amplitude spectra of a reference and a sample measurement obtained by performing a Fourier-transformation. The spectra correspond to the corrected time-domain signals.

2. Polymorphism of nifedipine: x-ray powder diffraction

The polymorphism of the nifedipine (NIF) forms prepared was controlled via x-ray powder diffraction (XRD) measurements. For the analysis a STOE STADI MP equipped with a Cu-K_{α}-Anode was used at room temperature. Diffraction patterns were recorded from 2θ = 5° to 2θ = 35° (step: 1.2, time/PSD step: 30 s) in transmission mode (Debye-Scherrer configuration).

The powder diffractograms are shown in Figure S2 and match with previously published data[2].

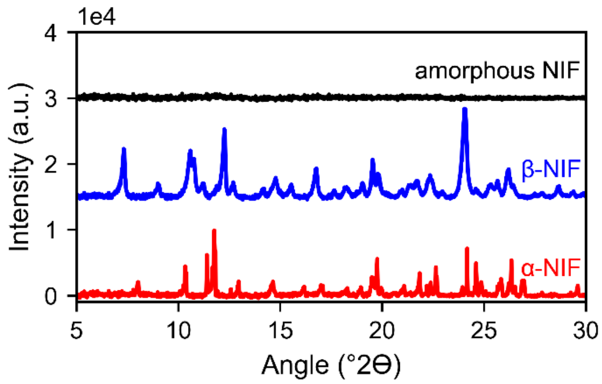

Figure S2. X-ray diffractograms of crystalline α- and β-NIF as well as unaltered amorphous NIF. For clarity, the diffractograms are shown with an offset of +1.5 (β-NIF) and +3.0 (amorphous NIF).

3. Influence of the T-Box on the THz-TDS measurements

The potential influence of the measurement platform on the THz-TDS measurements was controlled. For this, THz-TDS measurements of nitrogen with and without a built-in T-Box were conducted. The results are shown in Figure S3 and do not reveal any significant influence of the platform. Only a weak attenuation around 0.5 THz can be observed, which could be caused by the introduction of the measurement aperture.

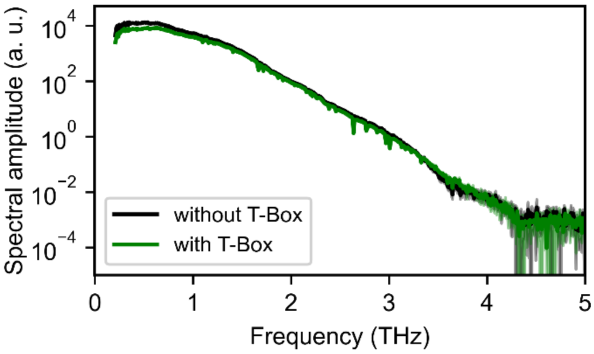

Figure S3. THz spectral amplitude with and without a built-in T-Box to estimate the influence on the THz-TDS results. The solid lines represent the mean spectral amplitude of five measurements (each with 100 averaged waveforms) and the corresponding standard deviation is shown as the shaded area around the curves. Note, that the sharp absorption lines are due to the low remaining humidity of the air insight the plastic box.

4. Advanced crystallinity analysis

For the advanced crystallinity analysis, the peak parameters of α- and β-NIF were extracted according to Ornik et al[3]. The identified peak properties are summarized in Table S1.

Table S1: Extracted Gaussian peak properties for the individual absorption peaks of α-NIF (α1- α3) and β-NIF (β1- β3). The properties include the peak amplitude (G), peak frequency position (ν_0) and peak width ($\Delta\nu$).

Peak	G (cm^{-1})	ν_0 (THz)	$\Delta\nu$ (THz)
α1	21.79	1.09	0.08
α2	49.37	1.20	0.06
α3	23.15	1.36	0.06
β1	2.73	0.95	0.05
β2	8.54	1.25	0.07
β3	7.57	1.50	0.08

To test whether the chosen approach also works for NIF under consideration of its polymorphism, i.e. by including the spectral features of α- and β-NIF, samples with known α-NIF content were studied. For the so-called physical mixtures, α-crystalline NIF was mixed with microcrystalline cellulose (Carl Roth GmbH + Co. KG) in different ratios and pressed to form tablets (prepared ratios: 0, 2.5, 5.8, 9.8, 17.9, 29.9 and 39.3 w% of α-NIF)[4]. Then the fit function (see main manuscript, Equation 1) was applied and the fit coefficients A and B were extracted. The two coefficients are related to the α-NIF (A) and β-NIF (B) content in the samples and are shown in Figure S4.

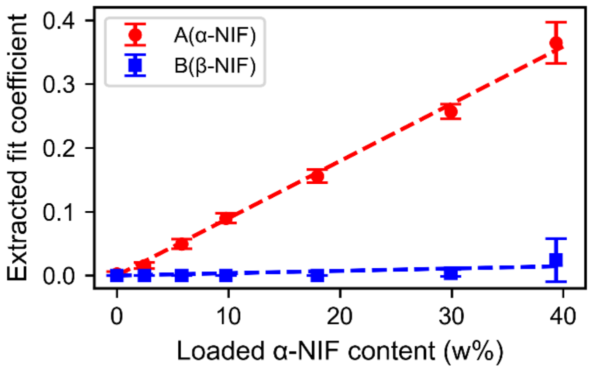

Figure S4. Extracted fit coefficients A and B as a function of the loaded α-NIF content in the physical mixtures. The error bars correspond to the standard deviation of the individually extracted fit coefficients for the three samples per NIF content. The red and blue dashed lines serve as a guide-to-the-eye.

As expected, with rising α-NIF content in the samples also the extracted coefficient A increases linearly. Furthermore, the extracted coefficient B, which relates to the β-NIF content, remains constant and close to zero. This is unsurprising, as no β-NIF was added to the physical mixtures. Thus, the applicability of the approach used can be confirmed, even though the peaks of the two polymorphs partially overlap.

References

1. Vázquez-Cabo, J. *et al.* Windowing of THz time-domain spectroscopy signals: A study based on lactose. *Optics Communications* **366,** 386–396; 10.1016/j.optcom.2015.12.069 (2016).
2. Gui, Y. *et al.* A Mechanism for Reversible Solid-State Transitions Involving Nitro Torsion. *Chem. Mater.* **32,** 7754–7765; 10.1021/acs.chemmater.0c02209 (2020).
3. Ornik, J. *et al.* Non-destructive crystallinity assessment of indomethacin in tablets made from smartFilms® using terahertz time-domain spectroscopy. *Scientific reports* **12,** 6099; 10.1038/s41598-022-10041-1 (2022).
4. Heidrich, L., Ornik, J., Keck, C. M., Castro-Camus, E. & Koch, M. Polyvinylpyrrolidone as co-inhibitor of crystallization of nifedipine in paper tablets. *International journal of pharmaceutics* **635,** 122721; 10.1016/j.ijpharm.2023.122721 (2023).
